# Game-based learning as training to use a chemotherapy preparation robot

**DOI:** 10.1177/10781552231181056

**Published:** 2023-06-08

**Authors:** Alexandra Garnier, Pascal Bonnabry, Lucie Bouchoud

**Affiliations:** 1 Pharmacy, 27230 Geneva University Hospitals, Geneva, Switzerland; 2 Institute of Pharmaceutical Sciences of Western Switzerland, School of Pharmaceutical Sciences, 27212 University of Geneva, Geneva, Switzerland

**Keywords:** Pharmaceutical technology, pharmacy, education, game-based learning

## Abstract

**Introduction:**

In 2015, our university hospital pharmacy acquired the PharmaHelp robot system to automate part of its chemotherapy production. Complex technical use, downtime periods, and insufficient training caused a drop in motivation and disparities in operators’ knowledge. We created a short, playful, standardized, gamed-based training program to address this, and evaluated its impact.

**Methods:**

Operators were classified as trainers or trainees according to their knowledge about Information and Communication Technologies. Before, after the training, and at 6 months (6M), their robot knowledge was assessed on a 0-24-scale, motivation and self-efficacy in using it on 0-to-100 scales. Pairwise comparison *t*-test with Bonferroni adjustment was used (*p* < 0.05 considered significant). Satisfaction was measured using a six-point Likert scale. Trainer/trainee teams participated in 2-hour training sessions with three games and a debriefing. For “Knowing the manufacturing steps,” cards with the steps were placed in the correct order. For “Knowing the criteria for using the robot,” teams guessed whether certain compounds could be used with the robot. For “Knowing how to handle production errors,” the answer to each error (taken from real-life issues) was selected from four options.

**Results:**

Participants (*n*  =  14) were very satisfied about sessions’ interactivity and playfulness. Knowledge improved from 57% pretraining to 77% (*p* < 0.005) to 76.6% (6M) (*p* < 0.05 compared to pretraining). Motivation and self-efficacy, respectively, improved from 57.6% to 86.6% (*p* < 0.05) to 70.4% (6M) and from 48.5% to 75.6% (*p* < 0.05) to 60.2% (6M) (*p* > 0.1 compared to pretraining) (*t*-test).

**Conclusions:**

This highly appreciated training program efficiently improved knowledge retention out to six months.

## Introduction

Our university hospital pharmacy produces approximately 17,000 chemotherapy preparations per year using CATO™ cytostatic production software. This enables the preparation of cytostatic drugs, or other substances, using gravimetric control carried out using electronic scales connected to a computer.^1^ However, cytotoxic compounding is a high-risk process with major consequences in case of errors. Human reliability can be suboptimal (resulting in preparation errors), and monitoring and verification may be limited. Moreover, there may be significant interoperator variability, for example, in preparation accuracy.^1^ As the number of preparations continues to increase in relation to the prevalence of cancers, difficulties maintaining adequate numbers of appropriately trained staff may appear. Automation is a potential solution to reducing the frequency of errors and improving preparation safety. Automation's main goals are avoiding errors (wrong drug, wrong dose); reducing interoperator variability; increasing accuracy, efficiency, and productivity; and improving traceability.^2^

In 2015, Geneva University Hospitals were the first in Switzerland to automate part of their cancer chemotherapy production by acquiring a PharmaHelp® robot (Fresenius Kabi). The objectives were improving the safety of these high-risk preparations for patients and staff, optimizing organizational efficiency, increasing productivity, and reducing the physical constraints on operators.^3^ Indeed, the repetitiveness and awkward postures associated with chemotherapy preparation within an isolator put operators at risk of musculoskeletal disorders.^4^ This complex, new technology was the subject of initial training sessions at acquisition, but these did not continue over time. These sessions were conducted following a conventional format consisting of trainees reading the recommended procedures, being shown the processes, and then repeating them themselves. However, operator hiring and departures gradually led to disparities in their training and knowledge. Concurrently, the robot's complex technical use and periods of machine downtime caused drops in motivation, which led to a return to using traditional nonautomated preparation methods. When the present study began, the robot was not being used to its full capacity, and the depreciation time for its cost was stretching out. To meet the need to upgrade and homogenize their skills, we decided to create a short, standardized, sustainable operator training program using innovative pedagogical approaches, such as game-based training. This paper discusses this training method's development and the evaluation of its effectiveness.

## Materials and methods

### Game-based training strategy

The Kern circle (a six-step approach to curriculum development) was used to design this training program concept.^5^ A problem was identified (step 1)—the variability in operators’ knowledge about the PharmaHelp® robot—and operator's needs were assessed (step 2)—the ability, as healthcare professionals specialized in pharmaceutical technologies, to be able to use the latest tools of their trade. These skills are particularly important when preparing cytotoxic drugs in cleanrooms because the standards of quality expected in Good Manufacturing Practices must be ensured. Operators are also sometimes the first to experience revolutionary technical evolutions in Information and Communication Technologies (ICT). Considering these points, understanding how operators act and react to these kinds of tools is a key means of supporting them through such transitions. Training program's general goals and specific objectives were then defined (step 3)—the general goals were the program's main learning orientations, which had to be achieved during the training sessions and had to relate to general needs. Our two general goals were developing operators’ understanding of the process (goal 1) and helping them acquiring the competencies to manage the robot (goal 2). Specific objectives flowed from general goals and described the learnings that operators were expected to have taken in by the program's end. Specific objectives are presented in Appendix 1. Bloom's taxonomy of the cognitive domain and Jewett's taxonomy of the psychomotor domain were used to draft our general goals and specific objectives.^6,7^

The pedagogical strategy was created from the content's specific objectives (step 4). Learning Designer®'s free online software was used to help build the training program.^8^ This made it possible to design a set of intended learning outcomes and, for each step, decide on guidance documents for the students, the type of learning to be achieved, its duration, group size, the necessary presence or not of a teacher, whether training was online or not and synchronous or not, and which resources needed to be linked to it. The training program comprised four parts: preparatory work (10 min), introduction (10 min), theory (45 min), and practice (45 min). For the preparatory work, participants read the institution's internal procedures about working with the robot and watched a video about the robot, at least 1 week before the training program.^9^ For the introduction, a mind map representing the training program's goals and the process for using the robot was created on Framindmap®.^10^ This useful, free online software highlights the semantics of the hierarchical links between different concepts and ideas in a very visual way.

The theoretical part of the training took place in a classroom and was divided into three 15-minute games, each one related to a specific objective. Each training session had two teams of two players. The trainer was a pharmacist who specialized in using the robot. For objective 1, the process's 16 steps were printed on cards that had to be put back into the right order. During the debriefing, the two teams compared and discussed their answers. For objective 2, fake Pokémon®-like cards presenting a compound and its specificities (stability, viscosity, usual dosage, etc.) were used. One team picked a card and read out the information written on it; the other team had to guess whether the compound could be used with the robot and why. During the debriefing, the trainer could correct any mistakes and summarize the criteria. For objective 3, we created a game inspired by the TV show “Who Wants to Be a Millionaire?” incorporating six problematic, real-life situations. Each problem was presented with four potential answers, which teams had to discuss and select the correct one (Appendix 2).

For the practical part, one team at a time (trainer/trainee) entered the cleanroom with the training pharmacist. Using an adapted version of the Peyton approach, the trainer became the trainee, and the trainee became the trainer (Table 1).^11^ The training pharmacist simply observed but was ready to help or give feedback. The trainer/trainee team was asked to prepare two bags of Ganciclovir (Cymevene®) 200 mg and a single chemotherapy of Cisplatin 39 mg. The team, thus, underwent a dose-banding exercise (the preparation of several bags with the same dosage, in advance, and with a long expiration date) and an individualized preparation exercise (preparation of one bag for a dedicated patient, with a short expiration date). Operators also experienced the difference between reconstituting a powder (adding a solvent to put the powder into suspension and injecting it into the bag) and using liquid chemotherapy. Training bags were not distributed to patients.

**Table 1.** The adapted Peyton approach.

	Peyton approach	Adapted Peyton approach
Demonstration	The trainer demonstrates the procedure without commenting on it	The trainee performs the procedure
Deconstruction	The trainer repeats the procedure, concurrently explaining every step	The trainee narrates every step of the procedure as they complete them
Comprehension	The trainee narrates every step while the trainer applies the procedure according to the trainee's instructions	The trainer corrects the procedure if necessary
Performance	The trainee performs the procedure themself	The trainer can intervene at any time and give feedback

At the end of the entire training program, there was a 10-minute debriefing where participants were encouraged to discuss what they had learned and to ask any final questions they had.

### Creation of the teams

A 2-minute survey (Appendix 3) was created to learn about operators’ usage of technologies. Their answers were interpreted using an ICT-usage table adapted to the healthcare environment.^12^ This classified operators into three categories: *resistant*, *functional*, or *expert* (Table 2). The questionnaire made it possible to identify those *resistant* operators who would need more support in their process of technologically mastering the robot and the *experts* who could, thanks to their advanced mastery of ICT in general, endorse the robot's use, and motivate their colleagues. The *functional* operators were autonomous in their use of ICT without being *experts* and were neither for nor against these technologies. Operators were divided into two groups, depending on their score, with the best scores placed in the trainer group and the lower scores in the trainee group. Each trainer was randomly paired with a trainee to constitute teams.

**Table 2.** Synthesis of the types of ICT users and their individual characteristics.

	Resistant	Functional	Expert
Number of usages	0–2	3–6	7–9
Usages	A few or no tools, basic usage	Tools and their functionalities, autonomous usage	Mastery of hardware and software
Logic of usage	Mediation	Utility	Identity
Significance of usages	Destabilization	Self-realization, personal self-management through increased efficiency and professional productivity	Projection in ICT, professional affirmation, ego reinforcement
Process of identity construction or reconstruction	Passive identity, ICT-generated identity rejection	Active identity, transition to another identity	Active identity, affirmation of their identity, the one generated by ICT

ICT: Information and Communication Technologies.

Usages: Daily chores, shopping, searching for information, desktop tools, emails, specific software, discussion forums, downloading, watching TV, phone calls, and preparing documents for work.

### Game-based training evaluation

In Kirkpatrick's Model of Training Evaluation, there are four levels of evaluation: level 1, *reaction*, is the degree to which participants found the training favorable, engaging, and relevant to their jobs; level 2, *learning*, is the degree to which participants acquired the intended knowledge, skills, attitude, self-efficacy, and commitment, based on their participation in the training; level 3, *behavior*, is the degree to which participants applied what they learned during their training once they were back at work; and level 4, *results*, is the degree to which targeted outcomes occurred because of the training program and the support and accountability package.^13^ The first two levels were used to assess the participants and evaluate our training program (step 6).

To evaluate level 1, participants answered a Likert-type satisfaction survey on a scale from 0–6 (agreement scale). To evaluate level 2, they answered a survey to identify operators’ baseline levels of knowledge about the robot in order to emphasize the identified critical points. Participants were asked to complete the survey at least one week before the training program. This survey of 10 questions scored from 0–24 examined operators’ knowledge about the robot itself and our hospital's process for using it (Appendix 4). Finally, a comment space allowed the operators to express themselves freely on the robot's advantages and points that needed to be improved. Two additional questions (using scales from 0–100) dealt with operators’ self-efficacy in their ability to use the robot and their motivation to do so. Immediately after the training program and at 6 months, participants had to answer the same questionnaire on their knowledge about the robot and their self-efficacy and motivation to use it.

### Statistical analysis

All the answers were collected and treated using Excel® software and analyzed using a pairwise comparison *t*-test with Bonferroni adjustment with *p* < 0.05 considered significant.

## Results

### Creation of the teams

Eighteen operators answered the questionnaire about their usage of ICT (Q#1). Scores ranged from 0–12 (mean  =  7.67, median  =  8.5, *SD*  =  3.76), and the operators with the best scores (min  =  9, max  =  12, mean  =  10.22, *SD*  =  1.20) were designated as trainers, and the others (min  =  0, max  =  8, mean  =  5.11, *SD*  =  3.72) were designated as trainees.

### Game-based training evaluation

**Figure 1.**
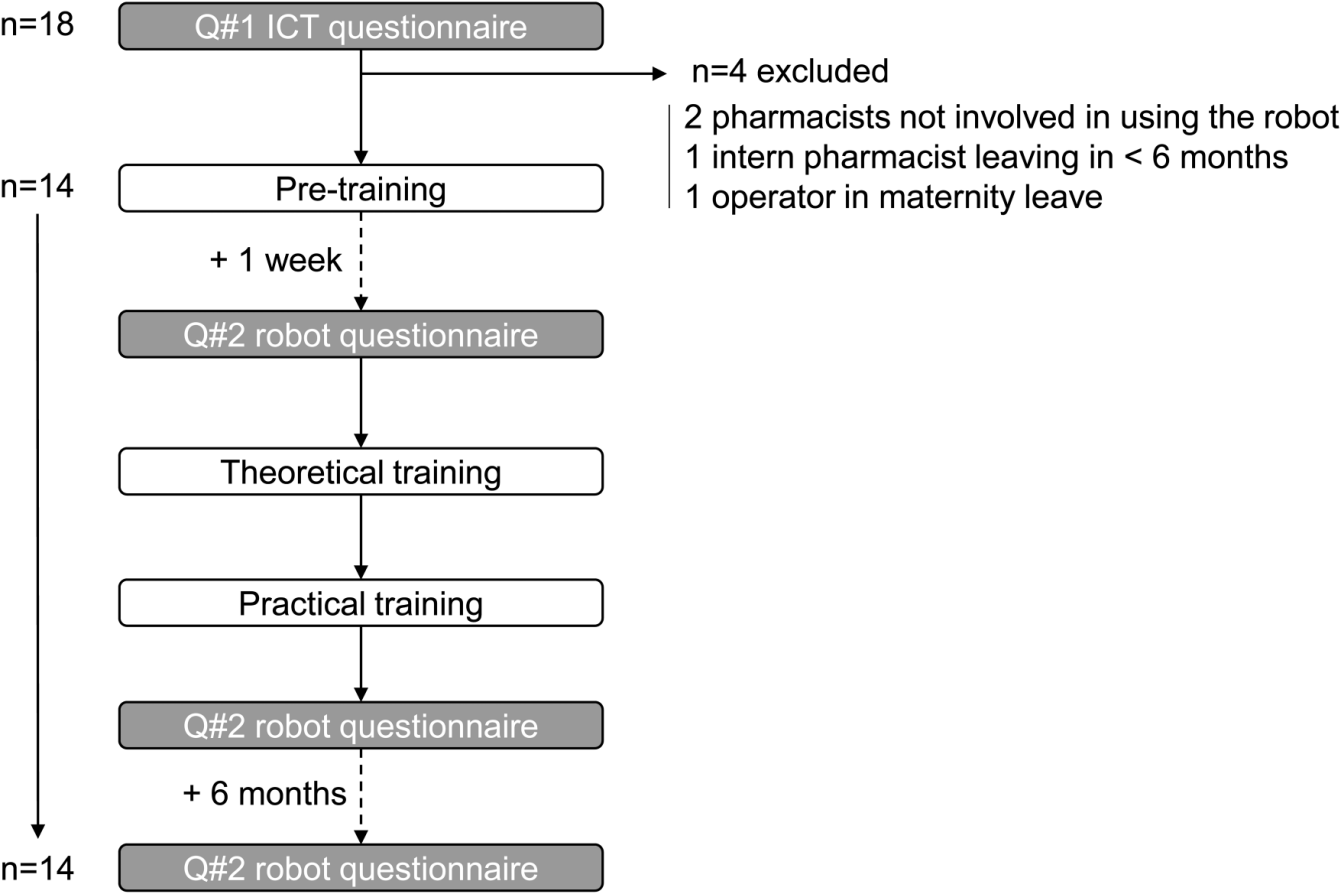


Out of the 18 operators who had answered Q#1, two were pharmaceutical technology pharmacists not involved in using the robot daily, one was an intern pharmacist planning to leave in less than 6 months, and one was an operator who left for maternity leave. Consequently, 14 operators participated in the training program and answered Q#2 before and immediately after training and at six months (Figure 1). The 14 operators participated in three sessions with two trainer–trainee teams and one session with just one team (seven teams in total). Thus, for the last training session, the sole participating team was not competing against another. Training took place during the afternoon with teams of trainer/trainee constituted according to the work schedule of the unit.

**Figure 2.**
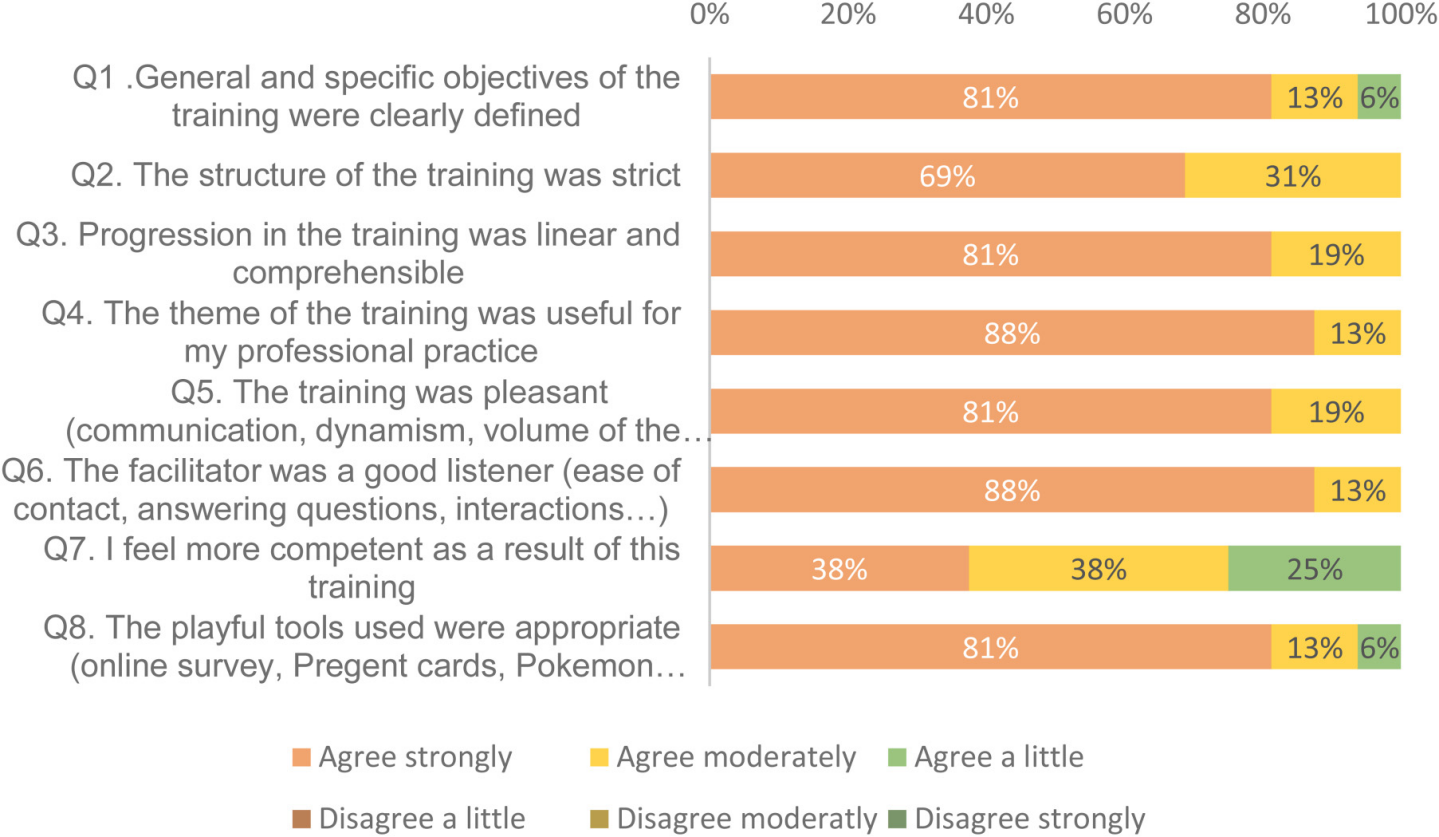
Post-training satisfaction.

The stacked bar chart in Figure 2 presents the participants’ overall very high satisfaction with their training—not a single *disagree* option was selected. Among the free comments, participants noted the program's interactivity *(“sharing our skills and experience with colleagues,” “makes you want to work in pairs,” “very pleasant; gives the opportunity to ask questions”)*, playfulness *(“good humor and friendliness,” “fun and dynamic training,” “we were captivated”),* and the appropriate format *(“the games allow an exchange and make us remember things more easily,” “good timing; the games are not too long and allow you to stay focused throughout the training,” “didactic and cover the main mistakes made in practice”).*

**Figure 3.**
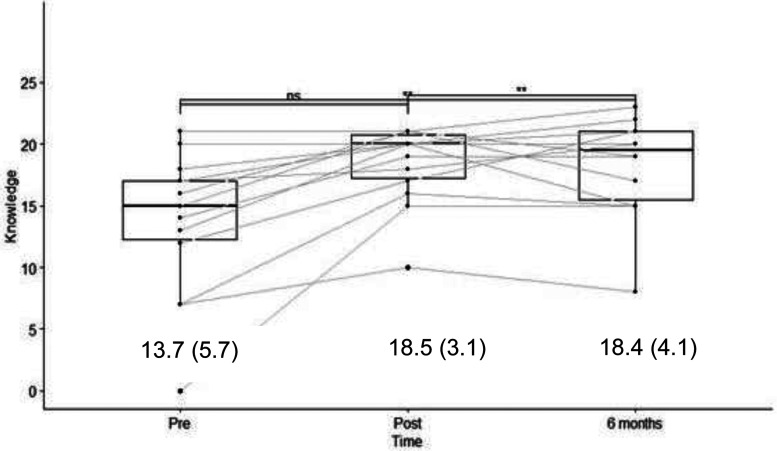
Results of the survey on knowledge about the robot, with results (score/24) and mean (SD).

**Figure 4.**
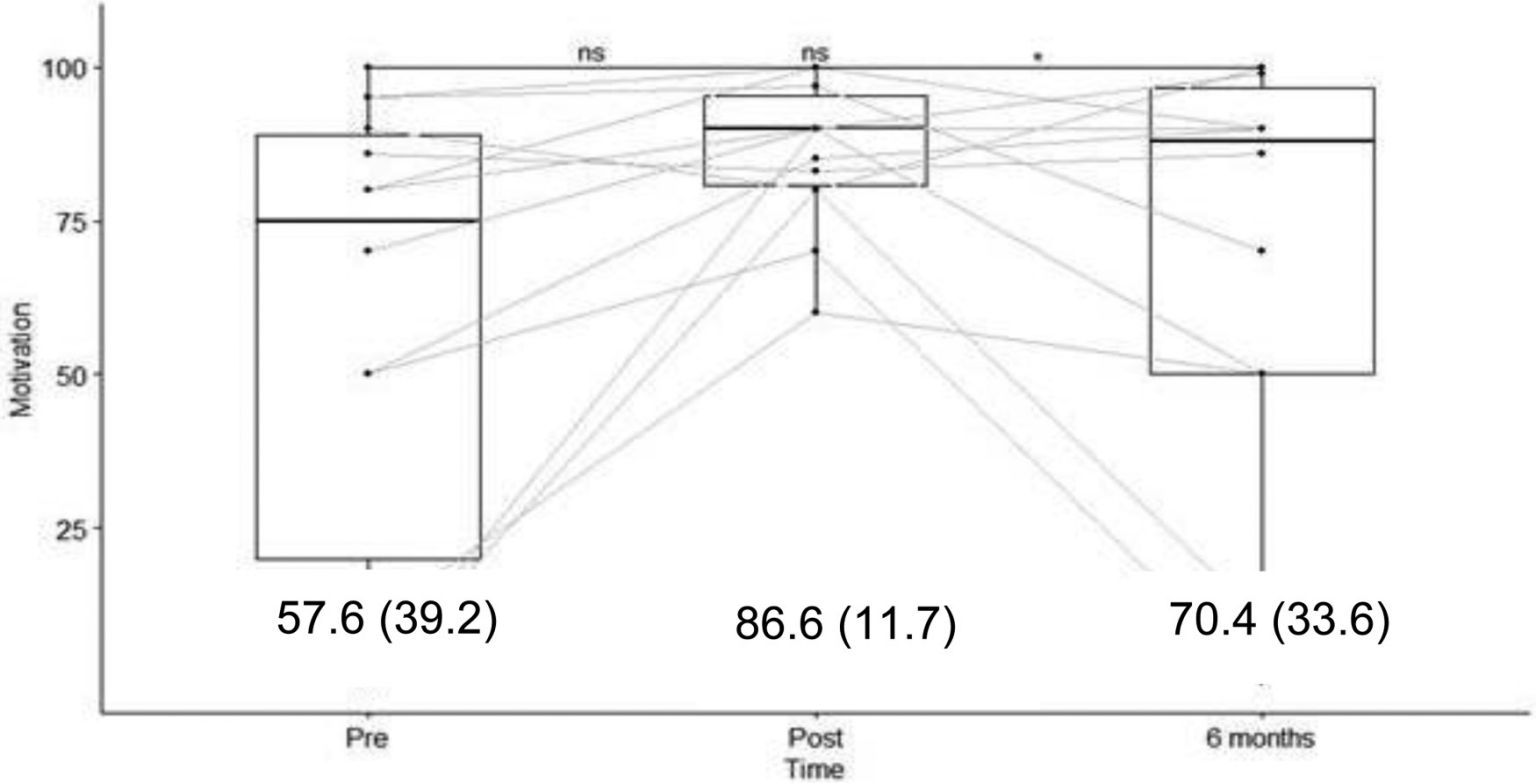
Results of the survey on motivation to use the robot, with results (score/100) and mean (SD). The bar in the box plot represents the median, whereas the mean (and standard deviation) is given by the numbers in the graph.

**Figure 5.**
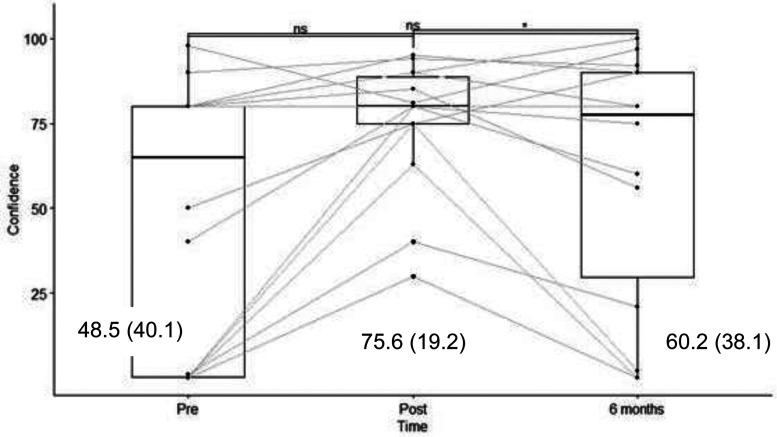
Results of the survey on self-efficacy in using the robot, with results (score/100) and mean (SD).

The knowledge scores were evaluated before and immediately after training with a maximum score of 24. Knowledge scores increased from a mean of 13.7  ±  5.7) to 18.5  ±  3.1) (*p* < 0.005) and stabilized at 18.4  ±  4.1) at seven months post-training (*p* < 0.005 compared to pretraining) (Figure 3). Translated into a percentage of correct answers, the change was from 57% to 77% immediately after the training and to 76.6% at six months. Motivation was evaluated on a scale from 0 to 100 and increased from a mean of 57.6  ±  39.2 to 86.6  ±  11.7 immediately after the training (*p* < 0.05); however, it decreased to 70.4  ±  33.6 at six months (*p* > 0.1 compared to pretraining) (Figure 4). Self-efficacy was evaluated on a scale from 0 to 100 and increased from 48.5  ±  40.1 to 75.6  ±  19.2 immediately after the training program (*p* < 0.05) but decreased to 60.2  ±  38.1 at six months (*p* > 0.1 compared to pretraining) (Figure 5). The before-to-after changes in these three criteria were statistically significant, but only the change in knowledge remained significant at six months (*t*-test).

## Discussion

### Training program’s impact

The program's 2-hour training sessions aimed to improve and standardize operators’ knowledge about the PharmaHelp® robot—a highly specialized, technical robot used to prepare chemotherapy preparations.

Before their training sessions, operators’ scores for knowledge, motivation, and self-efficacy were close to 50% (57%, 57.6%, and 48.5%, respectively), with huge variations (SD) between operators. These mean results reflect operators’ initial basic knowledge about the robot and their motivation to use it, factors closely linked to their self-efficacy in using it, which was not very high because it is technically complex to use, had significant downtime periods, and they had undergone insufficient training. After their training session, knowledge scores increased from 57% to 77%, and they were still at this level six months later. Another study assessing retained knowledge 1 month after a game-based learning experience in the same field went from 57% of correct answers pretraining to 88% immediately post-training and decreased to 80% at one month.^14^ Our program did not improve post-training knowledge results by as much, but at six months, they had not decreased. Although it is impossible to link the improvements in knowledge recorded to either the theoretical or practical parts of the training program, we can consider the mix of these two approaches to have had a positive impact on implanting that knowledge over time. The decrease in motivation and self-efficacy over time suggests that regular refresher sessions are necessary; however, it should be noted that our PharmaHelp® robot was little used during the six months post-training because of a breakdown (Figure 5).

Participants greatly appreciated the training sessions, and several comments mentioned how agreeable it was to train in pairs, even if participants were not used to working together regularly. Teamwork and the degree of collaboration were not assessed but probably contributed significantly to improving the training session's impact, as previously established in the literature.^15^ Working in pairs facilitates learning among peers and, as in a flipped-classroom model, the trainer has an accompanying role rather than a teaching role.^16^ This insight is useful as training in the healthcare field is tending toward individualization according to each learner's personal needs (the FAIR principle).^17^

Among the many learning methods (*ex-cathedra* classes, e-learning courses, small or large groups, etc.), educational games are an experiential learning method that can also contribute to improving student learning.^18^ In the context of game-based learning, “edutainment” helps to anchor training within a positive emotional environment. To improve pharmacy technicians’ skills, trainers have created sophisticated training methods such as serious games, virtual reality games, and escape games.^19^

Despite this growing worldwide movement toward using serious games and virtual reality, the training described in the present study was deliberately designed not to require internet access or powerful computers. Nevertheless, the concepts covered appealed to participants’ knowledge, know-how, and interpersonal skills.

The use of the Kern cycle—which is widely applied in the field of pedagogy—was a significant aid to the development of our ideas, especially in combination with the Bloom taxonomy for setting out our objectives. Learning Designer® software was useful for organizing timings and integrating training sessions into operators’ daily work schedules. Indeed, fitting 2 hours of training into operators’ heavy workloads was a fundamental challenge of this study. Chabrier et al. also considered it essential to design games in which the time required remains realistic in relation to participants’ constraints.^20^

The time spent preparing and setting up the training sessions was not specifically recorded but was consequent. The seven sessions themselves took 14 hours, which should be added to the time required to develop the training program itself, leading to a probable total of more than 100 hours. This was possible as the work was performed as part of a PhD thesis, with the doctoral student involved in 100% of the project. Other considerations should be made if this kind of training program were to be developed by pharmacists involved in day-to-day pharmacy operations.

### Limitations

The present study's limitations include its small sample size (14 operators) and the low number of questions (10) used to survey their knowledge. Moreover, how they applied the competencies learned during their training was not assessed over time. Further studies could investigate operators’ skills in using the robot immediately before and after the training session.

## Conclusion

We created a short, playful training session for a complex tool, one which can be applied to every new operator in the team and which demonstrated a good level of retained knowledge at six months. The time needed to develop and implement the training program was consequential but beneficial to the pharmacy. More studies comparing game-based training and traditional learning are necessary in the field of pharmaceutical technology.

## Appendix

### Appendix 3. Questionnaire on ICT use.

**Table:**

My initials are: ………….. My age is: □ Less than 30 years old □ From 30 to 34 years old □ From 35 to 39 years old □ From 40 to 44 years old □ More than 45 years old My job is: □ Pharmacist □ Pharmacy technician □ Pharmacy technician helper □ Intern I have been working here for: □ Less than 1 year □ Between 1 and 4 years □ Between 5 and 9 years □ Between 10 and 14 years □ More than 15 years I work: □ 100% □ Part-time (please note the %) At home, I use my computer for: □ Daily chores (paying bills, voting, online administration, etc.) □ Shopping, buying/selling □ Searching for information □ Using Microsoft Office tools (writing a letter in Word, keeping accounts in Excel, etc.) □ Electronic messaging □ Specialized software (accounting, etc.) □ Discussion forums □ Downloading □ Watching television □ Live chats, making calls □ Preparing documents for work □ Other (please specify) I own: □ A smartphone □ A tablet computer, iPad □ An mp3 player, iPod □ A classic cellphone □ A laptop computer □ Other (please specify) At home: □ I surf on these websites: ………………………………………………………….. □ I use these apps: ……..………………………………………………………………. □ I watch: ……………………………………………………………………………………. □ I work with these software packages: …………………………………………………….

### Appendix 4. Questionnaire on knowledge about the robot.

**Table:**

My initials are: ………….. How many different active substances can I use during the same run? A prescription canceled automatically on Cytotrace cancels the order on PharmaHelp. True or False? How many of each of these products do I need to use for a run? □ Yellow rubbish bin: ………. □ Sedimentation Petri dishes: ………. □ Sedimentation gloves dishes: ………. □ Little sterile fields: ………. □ Big sterile fields: ………. □ Pair of size 8 gloves: ………. Whatever the compound is, I should always put an air needle in the vial. True or False? If False, explain why. I can place my syringes anywhere on the carousel. True or False? If False, explain why. I can place my vials and bags anywhere on the rack. True or False? If False, explain why. The robot is cleaned: (pick the correct answer): □ Every day □ Once a week □ Twice a month □ Once a month □ Every three months □ Every six months □ Once a year Number these preparation steps in the correct order from 1 to 8. □ Process □ Pre-process □ Post-process □ IV-line purge □ Release of the bags □ Writing batch numbers in the PharmaHelp software □ Starting the run □ Sending the order to the robot If the “Hand Captor Laser” error message appears, what should you do? More than one correct answer is possible. □ I check that both of the isolator doors are closed □ I stop the run □ I reinitialize the robot □ I clean the isolator □ I take the sleeves out of the robot □ I inform a pharmacist if the error message remains The choice of the use of a molecule by the robot is made according to which criteria? More than one correct answer is possible. □ The volume to withdraw from the vial □ The price of the vial □ The compound’s chemical properties □ The level of risk for the operator On a scale from 0 to 100, my self-efficacy/confidence in using the robot is: …………… On a scale from 0 to 100, my motivation to use the robot is: …………… If you wish to add any further comments, please do so here.
